# Hepatitis C Direct Acting Antivirals and Ribavirin Modify Lipid but not Glucose Parameters

**DOI:** 10.3390/cells8030252

**Published:** 2019-03-15

**Authors:** Mary-Anne Doyle, Chrissi Galanakis, Erin Mulvihill, Angela Crawley, Curtis L. Cooper

**Affiliations:** 1 Division of Endocrinology & Metabolism, Department of Medicine, University of Ottawa, Ottawa, ON K1H 8L6, Canada; madoyle@ohri.ca; 2 Ottawa Hospital Research Institute, Ottawa, ON K1H 8L6, Canada; cgalanakis@ohri.ca (C.G.); acrawley@ohri.ca (A.C.); ccooper@toh.ca (C.L.C.); 3 Department of Biochemistry, Microbiology and Immunology, University of Ottawa, Ottawa, ON K1H 8M5, Canada; emulvihi@uottawa.ca; 4 University of Ottawa Heart Institute, Ottawa, ON K1Y 4W7, Canada; 5 Division of Infectious Diseases, Department of Medicine, University of Ottawa, Ottawa, ON K1H 8L6, Canada

**Keywords:** viral hepatitis, cirrhosis, antiviral therapy, insulin resistance, lipid metabolism

## Abstract

Chronic hepatitis C (HCV) infection perturbs lipid and glucose metabolism. The influence of direct acting antiviral (DAA) treatment and ribavirin on these measures was evaluated. Furthermore, the effect of HCV cure on these parameters was assessed. Participants were allocated to one of three 12-week treatment groups: non-cirrhotic genotype 1a-paritaprevir/ritonavir/ombitasvir/dasabuvir (PrOD) plus ribavirin; non-cirrhotic 1b-PrOD; compensated cirrhotic 1a or 1b-PrOD plus ribavirin. Fasting insulin, glucose, lipid and apolipoprotein measures were assessed at baseline, Treatment Weeks 4 and 12, and 12 and 24 weeks post-dosing. Twenty-three of 24 participants achieved SVR (PP= 23/24, 96% SVR). Overall, total cholesterol, low-density lipoprotein cholesterol (LDL-C), and triglyceride levels all increased in treatment and post-dosing. However, LDL-C levels decreased during treatment in ribavirin recipients. Fasting glucose, insulin, and HOMA-IR were unchanged during treatment and 12 weeks post-treatment. By 12 weeks post-treatment, controlled attenuation parameter (CAP) scores, a measure of steatosis, increased from baseline (mean 30.3 ± 63.5, *p* = 0.05). This regimen was safe and highly effective and did not influence glucose metabolism. Ribavirin exposure may mitigate some on-treatment lipid changes. Further mechanistic studies are needed to understand how ribavirin impacts lipid pathways, as there could be therapeutic implications. The metabolic pathophysiology of increased CAP score with HCV treatment requires explanation.

## 1. Introduction

Chronic hepatitis C affects 2–3% of the world population and is a leading cause of cirrhosis, hepatocellular carcinoma, and liver transplant [1]. More than 350,000 deaths per year are attributable to hepatitis C (HCV)-related complications [1]. There is evidence that HCV perturbs the metabolic milieu, which in turn influences the risk for complications and response to HCV antiviral treatment. Insulin resistance and type 2 diabetes are associated with an increased risk of hepatocellular carcinoma (HCC) [2], higher transplant complication rates [3], accelerated liver fibrosis [4], and possibly increased morbidity from cardiovascular and metabolic complications [5]. Impaired insulin sensitivity is associated with diminished antiviral treatment outcomes with interferon-based regimens [6,7].

Achieving an HCV cure may influence glucose homeostasis. Observational studies have demonstrated improved insulin sensitivity and reduced incidence of type 2 diabetes in patients who achieved sustained virological response (SVR) following treatment with interferon and ribavirin [6,8,9,10]. Successful viral clearance following treatment with telaprevir, interferon, and ribavirin was associated with lower homeostatic model assessment-insulin resistance (HOMA-IR) in genotype 1 patients (HOMA-IR SVR: 0.61 vs. non-responders: 1.15, *p* < 0.05) [11].

HCV suppression and cure also influence lipid levels. The HCV life cycle is dependent on the very-low-density lipoprotein (VLDL) pathway. Viral replication involves the formation of complexes termed lipoviral particles resulting in decreased secretion of VLDL [12]. The assembly of these lipoviral particles is believed to facilitate binding with low-density lipoprotein cholesterol (LDL-C) receptors and is considered a mechanism by which HCV gains entry to the hepatocyte [13]. Numerous studies have demonstrated lower total cholesterol, triglycerides (TG), high-density lipoprotein cholesterol (HDL-C), and LDL-C levels in patients with chronic HCV infection [14,15,16]. Lower lipid levels correlate with higher SVR with interferon-based HCV antiviral treatment [14,15,16,17]. Successful treatment of HCV with interferon and ribavirin is also associated with the reversal of hypolipidemia, and in some cases, lipid levels may increase to levels associated with increased cardiovascular risk [18,19].

Treatment of HCV has advanced dramatically in recent years with direct acting antivirals (DAAs) resulting in shorter treatment duration, improved safety profile, higher tolerance, and higher SVR rates [20,21,22]. The effects of DAA HCV medications while on treatment and SVR once cured on the metabolic milieu are not well described. In one study of 40 HCV participants receiving 14 days of monotherapy with the protease inhibitor danoprevir, declines in serum HCV RNA and HOMA-IR correlated [23]. In contrast, Meissner et al. did not observe a change in HOMA-IR levels between baseline and 24 weeks post-treatment in recipients of sofosbuvir and ribavirin [24]. In another study, eradication of HCV with sofosbuvir was associated with a decline in HbA1c [25]. 

We evaluated the on-treatment effect of DAA HCV treatment and influence of achieving SVR on measures of glucose and lipid homeostasis. Study participants received an HCV DAA regimen consisting of an HCV protease inhibitor (paritaprevir boosted with ritonavir), an NS5a inhibitor (Ombitasvir), and a polymerase inhibitor (Dasabuvir) (PrOD), with or without ribavirin. The role of ribavirin was specifically addressed given the ongoing debate as to the pros and cons of this adjunct medication in DAA regimens [26]. Cirrhosis was also considered in our analysis given pre-existing evidence of perturbation of glucose and lipid metabolism in those with advanced liver disease.

## 2. Materials & Methods

Twenty-four HCV genotype 1a- or 1b-infected patients were included in this single-center, open-label pilot study [ClinicalTrials.gov (Identifier: NCT02734173)]. Approval for this study was obtained from The Ottawa Health Science Network Research Ethics Board (REB #2015-0305).

Participants were recruited from The Ottawa Hospital Viral Hepatitis Program (Ottawa, Canada) between July 2015 and April 2016. All were 18 years or older, planned to initiate HCV antiviral treatment, and provided signed informed consent to participate. Exclusion criteria included decompensated liver disease, HOMA-IR < 2.0, HIV seropositivity, and chronic HBV infection defined as hepatitis B surface antigen positivity. Use of immune-suppressing medications, active malignancy, diabetes, use of lipid lowering medications, familial lipid disease, pregnancy, breastfeeding, and ribavirin contraindication(s) were also exclusionary.

Participants who met the inclusion criteria were allocated to one of the following treatment groups based on genotype and presence of cirrhosis: non-cirrhotic genotype 1a-infected participants receiving PrOD plus ribavirin, non-cirrhotic genotype 1b-infected participants receiving PrOD, and compensated cirrhotic genotype 1a or 1b-infected participants dosed with PrOD plus ribavirin. Ribavirin was dosed by weight: 1000 mg if 70 kg or less and 1200 mg if greater, divided bid.

Participant demographics, HCV RNA level and genotype, mode of infection, length of time since infection, alcohol consumption, smoking, and history of illicit drug use data was collected at baseline. Blood samples were collected for HCV RNA analysis at baseline, Weeks 4 and 12, and 12 weeks and 24 weeks post-treatment.

Measures of fasting insulin, glucose, total cholesterol, HDL-C, LDL-C, TG, HbA1c, apoA1, apoA2, apoB, apoC2, apoC3, and apoE were performed at baseline, Weeks 4 and 12, and 12 and 24 weeks post-treatment. Patients were advised to fast prior to blood draws. HOMA-IR score was calculated as per the following: (glucose × insulin)/22.5. IR was defined as having an HOMA-IR >2. A cut-off of 2 was selected, as this is a recognized standard [27]. Furthermore, our investigator group believed that evaluating a study population with elevated HOMA-IR would be optimal to identify an HCV treatment effect, if present.

Steatosis and liver fibrosis can result as a consequence of metabolic dysfunction. Liver stiffness was determined by transient elastography (FibroScan^®^, Echosens SA, Paris, France) [28], performed at baseline, Week 12, and 12 weeks post-dosing. A recording over 12.5 kPa defined cirrhosis. Controlled attenuation parameter (CAP) was utilized as a measure of steatosis and/or inflammation within the parenchyma of the liver.

### Statistical Analysis

A danoprevir mono-therapy study identified a 1.6 +/− 1.1 decline in HOMA-IR over 14 days [23]. In HCV patients treated with interferon and ribavirin with or without metformin, a 21% decrease in HOMA-IR in metformin recipients versus 10% in the control group was noted in the first 12 weeks (absolute 1.0 mean change at 24 weeks post-HCV antiviral treatment) [29]. Based on these data, a crude sample size of 8 per group provided 80% power to detect a mean difference in HOMA-IR scores of 1.0 assuming a two-sided comparison, a variance of 0.5, and an alpha of 0.05. This did not account for analysis adjustments for potential confounders, as this was a pilot study.

Patient demographics, glucose, lipids, and apolipoprotein measures, grouped according to treatment arm and differences between baseline characteristics, were evaluated using chi-square and ANOVA at the *p* < 0.05 level. Fisher’s exact test was used for outcomes with fewer than 5 cases. Changes from baseline in HOMA-IR, lipid, and apolipoprotein parameters at Weeks 4 and 12 as well as 12 weeks post-treatment and 24 weeks post-treatment were assessed using linear mixed models or generalized linear mixed models with a logit link. Models consisted of fixed effects for visit, treatment group, and their interactions with time. Models of glucose measures were adjusted for baseline BMI, and models of apolipoproteins were adjusted for baseline viral load. Visit was treated as a categorical variable with baseline as the reference. Models included a random intercept for participant or a repeated statement to control for the clustering effects of time. The estimated mean at each study time point was also determined by the mixed models. Pairwise comparisons of the estimated means within each group with respect to baseline were performed with least significant difference tests in the mixed models. As the primary outcome, changes in HOMA-IR from baseline to 12 weeks post therapy were compared within treatment groups.

## 3. Results

### 3.1. Demographics and Baseline Characteristics

Twenty-four participants were enrolled in this study. Of 38 screen failures, 31 had HOMA-IR results <2. Baseline characteristics are outlined in Table 1. The participants were predominantly male with a mean age of 54 years (SD 11.6) and a mean BMI of 30.0 kg/m^2^ (SD 4.6). The most common mode of HCV exposure was former injection drug use (48%) followed by blood transfusion (17%). Nine non-cirrhotic genotype 1a-infected participants received PrOD plus ribavirin therapy. Eight non-cirrhotic genotype 1b-infected participants were dosed with PrOD without ribavirin. Seven compensated cirrhotic genotype 1a or 1b-infected participants received PrOD plus ribavirin therapy.

### 3.2. HCV Treatment Response

All participants cleared HCV RNA by Week 4 of treatment. Twenty-three of 24 participants achieved SVR12. One participant with detectable virus at the end of treatment was lost to follow-up prior to the visit occurring 12 weeks post-treatment. The SVR12 was 100% (8/8) with ribavirin-free regimens and 15/16 (94%, PP = 15/16, 94%) in ribavirin-containing treatments (*p* = 0.47). SVR12 was 7/7 (100%; PP = 7/7, 100%) in cirrhotic participants and 16/17 (94%, PP = 16/17, 94%) in those without cirrhosis (*p* = 0.51). Ribavirin dosage was reduced to 600 mg in three patients following treatment initiation. One patient had a serious adverse event unrelated to the study drug and discontinued therapy at nine weeks to avoid a drug–drug interaction with colchicine for a severe gout flare resulting in hospitalization. No other serious adverse events were reported during the study.

### 3.3. Metabolic Measures and HCV Treatment—All Patients

**Figure 1:**
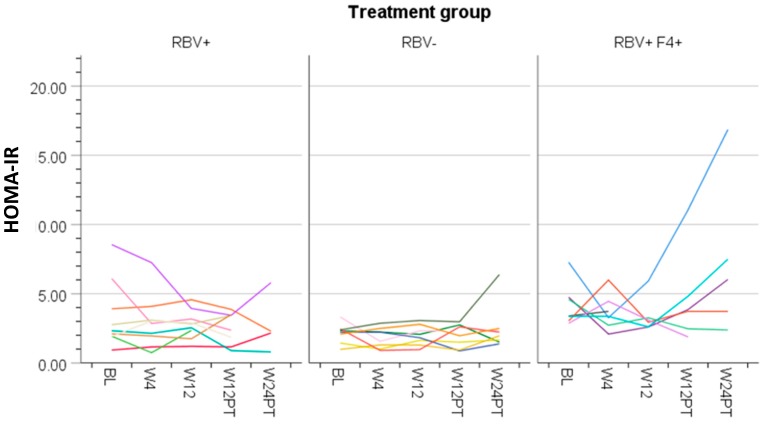
Homeostatic Model Assessment—Insulin Resistance (HOMA-IR) at each study time point (baseline, Week 4 of treatment, end of treatment at Week 12, 12 weeks after treatment completion, and 24 weeks after treatment completion) for each participant by treatment group (RBV+ = ribavirin containing hepatitis C (HCV) treatment in participants without cirrhosis, RBV− = ribavirin free HCV treatment in participants without cirrhosis, RBV+F4+ = ribavirin containing HCV treatment in participants with cirrhosis).

**Figure 2:**
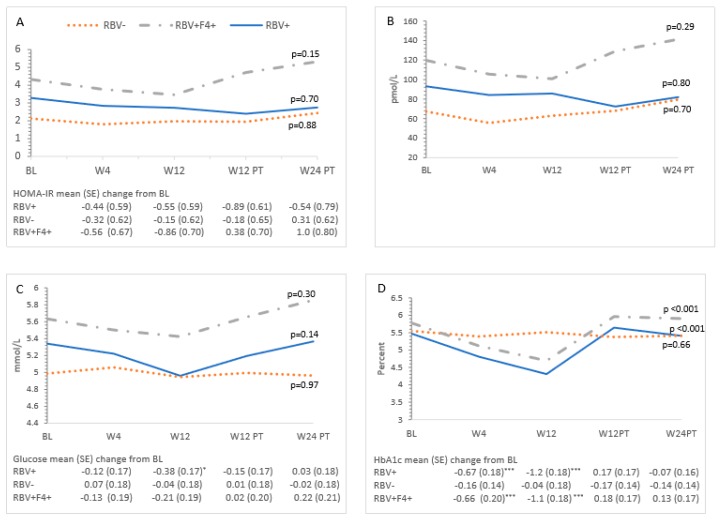
Changes in glucose measures over time and mean changes compared to baseline. (**A**) HOMA-IR, (**B**) insulin, (**C**) glucose, and (**D**) HbA1c. The estimated mean and the mean change from baseline at each study time point is from a linear mixed model adjusted for group, and the baseline BMI and their interaction with time is from a linear mixed model with a random effect for participant. HbA1c was adjusted for the effect of hemoglobin. All *p*-values were determined by the linear mixed model for the effect of time and group at each time point. Significant mean changes in the glucose measures compared to baseline at the *p* < 0.05 significance level were denoted by (*), *p* < 0.01 by (**), and *p* < 0.001 by (***). Missing data: baseline: HbA1c (n = 1); Week 4 (W4): HbA1c (n = 1); Week 12 (W12): insulin/HOMA-IR (n = 1), HbA1c (n = 3); 12 weeks after treatment (W12PT): glucose/HOMA-IR (n = 3), insulin (n = 2); HbA1c (n = 2); 24 weeks after treatment (W24PT): HOMA-IR (n = 7), insulin (n = 7), glucose (n = 4), HbA1c (n = 4).

**Figure 3:**
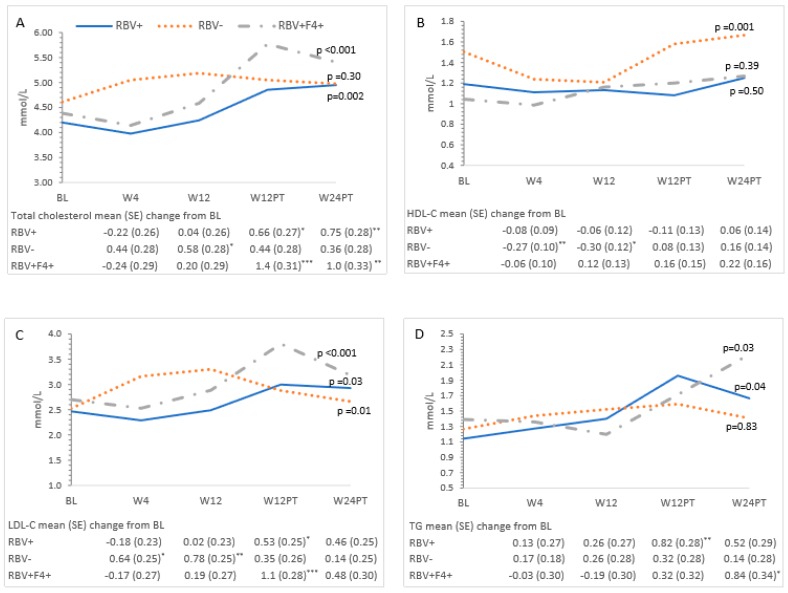
Changes in lipid measures over time and mean changes compared to baseline. (**A**) Total cholesterol, (**B**) high-density lipoprotein cholesterol (HDL-C), (**C**) low-density lipoprotein cholesterol (LDL-C), and (**D**) triglycerides (TG). The estimated mean and the mean change from baseline at each study time point is from a linear mixed model adjusted for group and its interaction with time from a linear mixed model with a random effect for participant. All *p*-values were determined by the linear mixed model for the effect of time and group at each time point. Significant mean changes in the glucose measures compared to baseline at the *p* < 0.05 significance level were denoted by (*), *p* < 0.01 by (**), and *p* < 0.001 by (***). BL = Baseline; W4 = Week 4 of treatment; W12 = Week 12 of treatment; W12PT = 12 weeks after treatment; W24PT = 24 weeks after treatment. Missing data: W12PT (n = 2) and W24PT (n = 4) for total cholesterol, triglycerides, and HDL-C; W12PT (n = 4) and W24PT (n = 4) for LDL-C.

**Figure 4:**
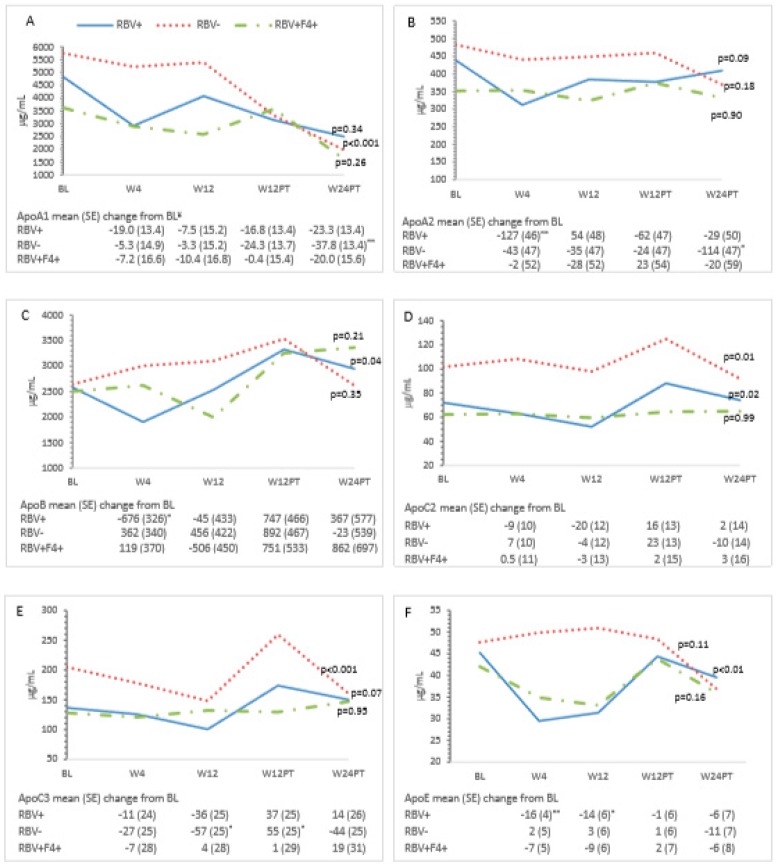
Changes in Apolipoproteins over time and mean changes compared to baseline. (**A**) apoA1, (**B**) apoA1, (**C**) apoB, (**D**) apoC2, (**E**) apoC3, and (**F**) apoE. The estimated mean and the mean change from baseline at each study time point is from the generalized linear mixed model adjusted for group, and baseline viral load and their interaction with time. All *p*-values were determined by the generalized linear mixed model for the effect of time and group at each time point. Significant mean changes in the apolipoproteins compared to baseline at the *p* < 0.05 significance level were denoted by (*), *p* < 0.01 by (**), and *p* < 0.001 by (***). ^¥^ Mean changes expressed as 10^2^. BL = Baseline; W4 = Week 4 of treatment; W12 = Week 12 of treatment; W12PT = 12 weeks after treatment; W24PT = 24 weeks after treatment. Missing data for all apolipoproteins apoA1–E at W12 (n = 1); W12PT (n = 2); W24PT (n = 4).

The means of the metabolic measures across the study period are presented in Table 2 and changes from baseline at each time point are reported in Figure 1, Figure 2, Figure 3 and Figure 4. Changes were observed for HbA1c and lipid measures across the study period (Table 2 and described below). Overall, glucose (*p* = 0.11), insulin (*p* = 0.42), and HOMA-IR (*p* = 0.32) did not change over time (Table 2, Figure 2A–C). Individual HOMA-IR trajectories over time are presented in Figure 1.

### 3.4. Ribavirin and Metabolic Measures 

Ribavirin-containing treatment was associated with a decrease in LDL-C at Weeks 4 and 12 (Figure 3C). This effect did not persist post-treatment. HbA1c decreased during treatment in the ribavirin-exposed group (Week 4: −0.50, 95% CI −0.91–−0.08; Week 12: −1.1, 95% CI −1.5–−0.68), but this change was not sustained post-treatment. No differences in HOMA-IR at each time point were observed in ribavirin-exposed participants compared to those who did not receive ribavirin. Compared to baseline, on-treatment decreases were observed at Week 4 in apoA2 and apoB or at Weeks 4 and 12 in apoE in ribavirin recipients (Figure 4A,B,F).

### 3.5. Cirrhosis and Metabolic Measures

Participants with cirrhosis had higher baseline insulin and HOMA-IR than non-cirrhotic patients in the RBV-sparing group (HOMA-IR mean difference 2.2, 95% CI 0.58–3.8, *p* = 0.01; insulin mean difference 52.5, 95% CI 8.5–96.4, *p* = 0.02). These measures remained unchanged from baseline while on treatment and 12 weeks following dosing in the overall study population as well as in cirrhotic and non-cirrhotic participants. Twenty-four weeks post-treatment, an increase in insulin (mean change 70.3, 95% CI 24.0–116.6) and HOMA-IR (mean change 2.9, 95% CI 1.2–4.7) was noted in cirrhotic participants, which was not observed in non-cirrhotic participants. When the results of a single outlier were removed, this finding was no longer present (Figure 1). Participants with cirrhosis had similar trajectories of HbA1c and lipid measures compared to those in the non-cirrhotic ribavirin-containing treatment group (Figure 2D and Figure 3A–D).

apoA1, apoA2, and apoE were lower at baseline in the cirrhotic participants compared with non-cirrhotic participants (Figure 4A,B,F). There were no observed changes from baseline in mean glucose and apolipoprotein measures among cirrhotic patients over the study period.

### 3.6. Liver Assessment

**Figure 5:**
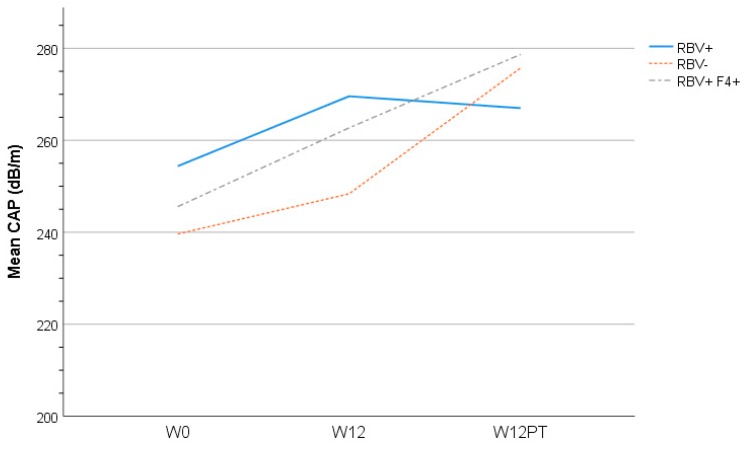
Mean controlled attenuation parameter (CAP) score at each study time point (baseline, end of treatment at Week 12 (W12), 12 weeks after treatment (W12PT) completion) by treatment grouping (RBV+ = ribavirin containing HCV treatment in participants without cirrhosis; RBV− = ribavirin free HCV treatment in participants without cirrhosis; RBV+F4+ = ribavirin containing HCV treatment in participants with cirrhosis).

Twelve weeks post-treatment, a mean increase from baseline in the controlled attenuation parameter (CAP) was observed (30.3 ± 63.5, *p* = 0.05) (Figure 5). No differences in CAP score were observed in the treatment group (*p* = 0.94). Overall mean liver stiffness scores did not change from baseline at 12 weeks post-treatment (−0.61 ± 5.9 kPA, *p* = 0.64). No differences were observed in liver stiffness over time by treatment group (*p* = 0.96) (data not shown). 

## 4. Discussion

An association between chronic HCV infection and impaired glucose and lipid metabolism has been extensively described in the literature [14,15,16,30,31,32,33]. Although the exact mechanisms by which HCV interferes with these metabolic pathways has not been fully established, there is evidence to suggest that viral clearance achieved with interferon-based HCV treatment may perturb these metabolic measures [8,9,10,18,19,25]. In contrast, there has been minimal evaluation of the metabolic effects of interferon-free DAA treatment or the specific effects of ribavirin while in therapy or after SVR. We present a prospective comprehensive analysis of metabolic findings of individuals undergoing interferon-free, DAA treatment (with and without ribavirin) as well as the post-SVR outcomes of HCV genotype 1 infection.

We observed no consistent on-treatment or post-SVR change in measures of glucose homeostasis. HOMA-IR did not improve from baseline during the treatment phase or post-DAA dosing in the overall study population or any subgroup (Table 2, Figure 1 and Figure 2A). The latter is consistent with results from a Messiner et al. study in which no difference in HOMA-IR between baseline, on-treatment and the post-treatment phase were observed in patients treated with an IFN-free regimen [24]. This challenges the hypothesis that HCV clearance may reduce insulin resistance and/or reduce the risk of developing diabetes [8,9,10]. As suggested in Figure 1, it is possible that the complex interactions between liver fibrosis status, HCV RNA clearance, and glucose-insulin homeostasis may result in heterogeneous short- and long-term outcomes regarding diabetes risk.

Our evaluation suggests a correlation between the degree of liver fibrosis and insulin resistance (see Figure 2 baseline results). Insulin resistance is a critical factor in promoting the progression of hepatic fibrosis. This is thought to be mediated via the direct effect of insulin on hepatic stellate cells [27]. Insulin resistance is a common and well-known feature of cirrhosis irrespective of etiology and an important contributor to hepatogenous diabetes [34,35]. 

There was a difference in HbA1c measures based on ribavirin exposure. A decrease in HbA1c levels was observed in ribavirin recipients during the treatment phase. As there were no differences in fasting glucose or insulin levels at these time points, HbA1c may not provide a true reflection of glycemic control in this group. Ribavirin reduces erythrocyte lifespan and in turn may lead to falsely low HbA1c levels [36,37]. It is unclear if other studies have considered the ribavirin effects on hemoglobin when describing improvements in HbA1c levels while on therapy [10,24,38,39]. One recent evaluation specifically indicated that HbA1c was not measured in ribavirin-exposed participants for a minimum of 3 months post-treatment due to the known hemolytic effects of this medication [25]. This group reported that HCV eradication with a sofosbuvir-based regimen resulted in a decrease in HbA1c levels (pre-treatment 6.66 +/− 0.95 versus post-treatment 6.14 +/− 0.65, *p* < 0.005).

In addition to evaluating measures of glucose homeostasis, we also assessed the lipid profile. As described previously, viral clearance was associated with increased on-treatment and post-treatment total cholesterol, LDL-C, and TG levels (Table 2, Figure 3A,C,D) [18,19,24]. The presence of cirrhosis did not affect lipid levels during or after treatment. Differences in the direction of lipid changes during treatment were observed based on ribavirin exposure, suggesting a direct effect of this medication on lipid homeostasis. Specifically, reduced total cholesterol, LDL-C, and decreased apoB levels were noted in non-cirrhotic RBV recipients at Weeks 4 and 12 (Figure 3A,C and Figure 4C). Post-treatment, this RBV-specific lipid effect was no longer observed. While the observed overall on-treatment and post-SVR increase in total and LDL-C, as well as TG levels, are consistent with other studies, the marked on-treatment effect of ribavirin on the lipid profile is novel. This warrants further evaluation given the possible role in the management of dyslipidemia.

We conducted an intensive evaluation of apolipoproteins. The formation of HCV lipoviral particles (LVP) occurs within the endoplasmic reticulum at interfaces between lipid droplets and is closely intertwined with lipid and lipoprotein metabolism [40]. As such, apolipoproteins are essential regulators of lipid metabolism and play an important role in the HCV life cycle [41]. apoB forms the protein backbone for the formation of TG lipoproteins including chylomicrons and VLDL as well as post-hydrolysis LDL. In contrast to apoB, other apolipoproteins including apoA1, apoC2, apoC3, and apoE are easily disassociated and can be exchanged between different classes of lipoproteins and the surface of HCV LVPs [41]. apoE association with HCV lipoviral particles enhances infectivity [13,42,43,44,45]. We observed a decline in apoE during the 12-week treatment phase in ribavirin-exposed participants. This was similarly described by Younossi et al. This further suggests a ribavirin-specific effect on lipid homeostasis. 

The downregulation of apoA1 has been associated with decreases in HCV RNA levels and as such has been implicated in viral replication [46]. Our study demonstrated decreased apoA1 levels in non-cirrhotic, RBV unexposed treatment recipients (Table 2, Figure 4A). This was in contrast to Younossi et al. where HCV treatment did not influence apoA1 levels. Although associated with low apoB levels, the role of apoB in HCV assembly is unclear [31,43,47,48]. apoB levels increased from baseline to 24 weeks after treatment in patients treated with RBV irrespective of the degree of liver fibrosis (Table 2, Figure 4C). This is in contrast to the study by Younossi et al. where treatment did not influence apoB levels. apoC2, an activator, and apoC3, an inhibitor of lipoprotein lipase activity, have been implicated in HCV infection via their modulation of the LVP catabolism. Lipoprotein lipase activity has been shown to inversely correlate with HCV RNA levels [49,50,51], and low apoC2 levels correlate with increased HCV infection and more advanced liver disease [50]. Consistent with other studies, we demonstrated an increase in apoC2/C3 levels post-treatment in non-cirrhotic patients irrespective of ribavirin exposure and provide further evidence that more advanced liver disease is associated with low levels of these apolipoproteins pre- and post-treatment (Table 2, Figure 4D,E).

CAP score, a measure of liver steatosis, was noted to increase from baseline at the end of treatment and again 12 weeks after treatment when SVR was achieved (Figure 5). This finding was consistent irrespective of the presence or absence of cirrhosis or RBV exposure. The explanation of this finding is unclear. The relationship between lipid profile perturbation and liver steatosis resulting from HCV RNA clearance requires further evaluation. This increase in CAP score did not correlate with the fibrosis score, which remained unchanged from baseline—at least during the short period of post-treatment follow-up.

There are limitations requiring consideration in this exploratory study. As all but one participant achieved a SVR12, it was not possible to compare metabolic outcomes between those cured and treatment failures. This was a non-randomized, open-label study and as such is subject to selection bias. Given the small sample size, it was not possible to adjust for all potential confounders, and the potential for Type I and II statistical error is acknowledged. Our study was powered to detect differences in HOMA-IR of 1.0. We were limited in detecting smaller changes in this primary outcome. Some evaluations have suggested that the most marked improvements in insulin sensitivity and glucose metabolism are achieved in patients with higher baseline HOMA-IR levels and established diabetes, respectively [25,38,39]. Our study population was characterized by relatively low HOMA-IR levels at baseline compared to these studies.

## 5. Conclusions

Exposure to DAA and ribavirin may influence lipid and apolipoprotein, but not glucose, parameters during HCV treatment. Furthermore, an HCV cure with DAA treatment results in increased lipid levels. Our study suggests that ribavirin exposure may play a role in mitigating some of the on-treatment lipid changes observed as HCV is cleared while on HCV treatment. Hepatic steatosis may also be affected by the clearance and cure of HCV.

## Figures and Tables

**Table 1:** Patient characteristics at baseline.

Characteristic	All Patients (n = 24)		RBV+ (n = 9)		RBV− (n = 8)		RBV+F4+ (n = 7)		*p*-Value **
	*n*	*%*	*n*	*%*	*n*	*%*	*n*	*%*	
Age *	54 (11.6)	-	53 (9.9)	-	51 (12.7)	-	59 (12.2)	-	0.36
Male	17	71	7	79	5	63	5	71	0.79
Genotype									
1a	13	54	9	100	-		4	57	<0.001
1b	11	46	-	-	8	100	3	43	
Race									
White	19	79	9	100	4	50	6	86	0.02
Indigenous	4	17	-	-	4	50	-	-	
South East Asian	1	4	-	-	-	-	1	14	
Years infected *	31 (11.3)	-	33 (12.1)	-	30 (7.9)	-	31 (16.3)	-	0.91
History HCV treatment	4	17	2	22	1	13	1	14	0.85
Fibrosis score * (kPa)	9.7 (6.6)	-	6.9 (2.5)	-	5.9 (2.0)	-	18.3 (6.3)	-	<0.001
F0-F1	12	50	6	67	6	75	-	-	<0.001
F2	3	13	1	11	2	25	-	-	
F3	2	8	2	22	-	-	-	-	
F4	7	29	-	-	-	-	8	100	
Controlled Attenuation Parameter (CAP) score *	246 (69.1)	-	254 (78.4)	-	240 (71.9)		242 (64.2)		0.91

* Mean (SD). ** *p*-values reflect a comparison between non-cirrhotic ribavirin HCV treatment recipients (RBV+), non-cirrhotic non-ribavirin HCV treatment recipients (RBV−), and cirrhotic ribavirin HCV treatment recipients (RBV+F4+).

**Table 2:** Estimated mean of metabolic parameters across the study period.

Metabolic Parameter	Estimated Mean (95% CI) *					*p*-Value
	Baseline	Week 4	Week 12	12 Weeks Post-Treatment	24 Weeks Post-Treatment	
HOMA-IR	3.2 (2.6–3.9)	2.8 (2.2–3.4)	2.7 (2.1–3.4)	3.0 (2.3–3.7)	3.5 (2.7–4.3)	0.32
Insulin	93 (76–111)	82 (65–99)	83 (66–101)	90 (72–108)	101 (80–122)	0.42
Glucose	5.3 (5.1–5.5)	5.3 (5.1–5.4)	5.1 (4.9–5.3)	5.3 (5.1–5.5)	5.4 (5.2–5.6)	0.11
HbA1c *	5.6 (5.4–5.8)	5.1 (4.9–5.3)	4.8 (4.6–5.0)	5.7 (5.5–5.8)	5.6 (5.4–5.8)	<0.001
Cholesterol	4.4 (4.1–4.7)	4.4 (4.1–4.7)	4.7 (4.4–5.0)	5.2 (4.9–5.5)	5.1 (4.8–5.4)	<0.001
HDL-C	1.2 (1.1–1.4)	1.1 (0.92–1.3)	1.2 (0.98–1.4)	1.3 (1.1–1.5)	1.4 (1.2–1.6)	0.01
LDL-C	2.6 (2.3–2.8)	2.7 (2.4–2.9)	2.9 (2.6–3.2)	3.2 (2.9–3.5)	2.9 (2.6–3.2)	<0.001
TG	1.3 (0.96–1.6)	1.4 (1.0–1.7)	1.4 (1.1–1.7)	1.8 (1.4–2.1)	1.8 (1.4–2.1)	0.01
apoA1	4647 (3290–6566)	3534 (2685–4654)	3842 (2889–5111)	3343 (2775–4026)	1997 (1436–2777)	0.002
apoA2	421 (376–471)	365 (321–415)	382 (337–433)	402 (356–453)	369 (321–424)	0.27
apoB	2575 (2329–2846)	2466 (2180–2790)	2503 (2101–2983)	3370 (2902–3913)	2962 (2396–3662)	0.03
apoC2	79 (67–90)	78 (67–89)	70 (58–81)	93 (81–104)	77 (65–90)	0.004
apoC3	153 (128–183)	139 (115–169)	125 (101–156)	180 (152–213)	153 (127–184)	0.02
apoE	45 (39–51)	38 (32–44)	39 (33–44)	46 (40–51)	37 (31–44)	0.003

* The estimated mean of the metabolic parameters at each study visit is adjusted for treatment group, baseline BMI, and baseline viral load by the linear or generalized linear mixed model. The *p*-value reflects the overall effect of time on the outcome across all study visits and study groups as estimated by the linear and generalized mixed models. ** HbA1c was also adjusted for the effect of hemoglobin.

## List of Abbreviations

HCV	Hepatitis C
RBV	Ribavirin
PrOD	Paritaprevir/ritonavir/ombitasvir/dasabuvir
CAP	Controlled attenuation parameter
SVR	Sustained virologic response
LDL-C	Low-density lipoprotein cholesterol
HOMA-IR	Homeostatic model assessment—insulin resistance
HCC	Hepatocellular carcinoma
VLDL	Very-low-density lipoprotein
TG	Triglycerides
HDL-C	High-density lipoprotein cholesterol
DAA	Direct acting antivirals
RNA	Ribonucleic acid
REB	Research Ethics Board
kPa	Kilopascal
BMI	Body mass index
IFN	Interferon
HbA1c	Glycated hemoglobin
LVP	Lipo-viral particles
